# Custom Design and Analysis of High-Density Oligonucleotide Bacterial Tiling Microarrays

**DOI:** 10.1371/journal.pone.0005943

**Published:** 2009-06-17

**Authors:** Gard O. S. Thomassen, Alexander D. Rowe, Karin Lagesen, Jessica M. Lindvall, Torbjørn Rognes

**Affiliations:** 1 Centre for Molecular Biology and Neuroscience (CMBN), Institute of Medical Microbiology, University of Oslo, Oslo, Norway; 2 Centre for Molecular Biology and Neuroscience (CMBN), Institute of Medical Microbiology, Oslo University Hospital, Rikshospitalet, Oslo, Norway; 3 Department of Informatics, University of Oslo, Oslo, Norway; Max Planck Institute for Evolutionary Anthropology, Germany

## Abstract

**Background:**

High-density tiling microarrays are a powerful tool for the characterization of complete genomes. The two major computational challenges associated with custom-made arrays are design and analysis. Firstly, several genome dependent variables, such as the genome's complexity and sequence composition, need to be considered in the design to ensure a high quality microarray. Secondly, since tiling projects today very often exceed the limits of conventional array-experiments, researchers cannot use established computer tools designed for commercial arrays, and instead have to redesign previous methods or create novel tools.

**Principal Findings:**

Here we describe the multiple aspects involved in the design of tiling arrays for transcriptome analysis and detail the normalisation and analysis procedures for such microarrays. We introduce a novel design method to make two 280,000 feature microarrays covering the entire genome of the bacterial species *Escherichia coli* and *Neisseria meningitidis*, respectively, as well as the use of multiple copies of control probe-sets on tiling microarrays. Furthermore, a novel normalisation and background estimation procedure for tiling arrays is presented along with a method for array analysis focused on detection of short transcripts. The design, normalisation and analysis methods have been applied in various experiments and several of the detected novel short transcripts have been biologically confirmed by Northern blot tests.

**Conclusions:**

Tiling-arrays are becoming increasingly applicable in genomic research, but researchers still lack both the tools for custom design of arrays, as well as the systems and procedures for analysis of the vast amount of data resulting from such experiments. We believe that the methods described herein will be a useful contribution and resource for researchers designing and analysing custom tiling arrays for both bacteria and higher organisms.

## Introduction

The availability of affordable custom-made expression arrays is increasing, and the feature number on oligonucleotide microarrays has increased remarkably during the last few years. Traditional Affymetrix GeneChip arrays focus on probing the coding sequences of known genes, and the probes usually only cover the annotated transcripts' 3′ end, hence much information regarding new transcripts (e.g. microRNAs, anti-sense transcripts and new genes), as well as splice variants of both known and unknown transcripts, are never found [1], [2]. Also, recent reports show that annotated genes tend to contain methylation sites with biased distribution towards the 3′ end. This bias in the expressed gene indicate that methylation might interfere with transcription initiation and termination [3], [4]. To address this problem, new microarray approaches that enable mapping of the total genome have emerged [5]. Tiling probes on the microarrays is one strategy that has been developed to completely cover areas of the genome [6]. For the majority of completely sequenced genomes no such arrays are currently on the market. Researchers therefore need to design the tiling array themselves. One great advantage of custom made arrays is that they enable total control over chip content with regard to probes for expression measurements, control probes and the distribution of probes over the array.

There are many aspects that have to be taken into consideration in order to achieve high quality data when designing microarrays; including probe density, probe-length, melting temperature, probe placement, strand coverage, cross-hybridization/probe-sequence complexity, probe uniqueness and control probes. The probe-specific aspects mentioned above make up a set of probe-properties. All probes on an array should ideally have approximately the same properties to ensure a constant probability of hybridization [7], the mean value of all these properties can be referred to as the consensus property. The ultimate, but impossible achievement, is to obtain dense coverage of an entire genome by probes with high consensus properties.

Today, several methods for the estimation of background signal level (sum of noise and non-specific hybridization) and data normalisation exist, but these are designed to work with commercial arrays (MAS 5.0, RMA, MBEI, and gcRMA) [8]–[11]. Such methods might rely on mismatch-probes [12] or assume that the majority of probes target coding regions, and are therefore often sub-optimal for non-standard custom arrays. Meanwhile, the more generally applicable analysis algorithm MAT (Model-based Analysis of Tiling-arrays) [13], originally designed for ChIP tiling arrays, would be sub-optimal for this study as it applies a 600 bp window which is far larger than the short transcripts targeted here (<60 nts). Other methods for dividing a transcriptome into discrete transcription segments involve different applications of hidden Markov models (HMMs), for instance the supervised Markov model framework of Du *et al.* [14]. One downside of HMM based methods is the need for a training set (generally originating from annotated regions of the genome) which necessarily guides the method towards the recognition of regions which are characteristically similar to the training set. Since a major goal of the approach presented here is to locate novel, short, differentially expressed transcripts in unannotated regions, a standard training set is not optimal. Finally, an HMM method which may successfully work on a single stressed or unstressed dataset will not simultaneously be applicable to data from a direct reference vs stress transcription comparison.

Present analysis methods for microarrays are mainly focused on known coding regions [8], [10], and researchers soon run into problems when trying to analyse signals from intergenic regions or un-annotated genomes, because of the difficulty in defining consistently expressed segments of the genome without the aid of an annotation. These problems can be addressed by applying the methods presented here, and the annotation-independent analysis method can be applied to any tiling array project, regardless of whether the investigated regions are coding or non-coding, and without the need of any genomic annotation or training set.

In this manuscript we present a novel design method for tiling arrays, here targeting prokaryotic genomes, but easily applicable to eukaryotic genomes as well. We present a novel normalization method suited to equidistantly or un-equidistantly distributed probes on tiling arrays. Additionally, we show how increased numbers of control probes, including random controls, can be used to assess the levels of non-specific binding and noise, which is always more or less of a problem with microarrays. Finally, we present two different analysis methods for genome-wide tiling array data, of which the latter is independent of annotations and training-sets.

## Methods

There are several important considerations regarding microarray design and analysis. Here we present a method for designing tiling arrays and methods for normalisation, background estimates/adjustments and data analysis of tiling experiments. As an initial project, two different prokaryotic genomes are used, the *E. coli* K12 MG1655 genome and *N. meningitidis* MC58 genome, respectively.

### Microarray design

Genomic coverage will always be a trade-off between probe-length, genome size and array feature number. The choices made here ensure coverage comparable to regular gene chips of all genes with a known function, as well as a very high coverage of the remaining genome. The arrays used in this project are the 280,000 feature NimbleExpress [15]–[17] custom arrays provided by Affymetrix, as this was the most reasonable choice when considering the feature number versus production cost. The oligo length was set to 25 nucleotides. The bacterial genomes and annotations of *E. coli* K12 MG1655 [GenBank:NC000913] and *N. meningitidis* MC58 [GenBank:NC003112] used for the probe design were downloaded from the NCBI ftp-site (24^th^ of May 2005). A basic tiling strategy places a probe at every Nth nucleotide (for some N where N<probe-length). Such an approach does not apply any probe-quality measures except for the widely used exclusion of repeat-elements from the target sequences (by using programs such as RepeatMasker [18] or Dust (Tatusov RL and Lipman DJ, unpublished). Use of probes covering repeat elements in the genome should be avoided because of the high risk of cross-hybridisation by similar probes with plural origin, generating meaningless data within these regions. If a more selective tiling approach is used, as described in this paper, it should be possible to choose a set of probes that are more homogeneous, reducing the noise that is otherwise introduced by significant probe-affinity differences.

**Figure 1:**
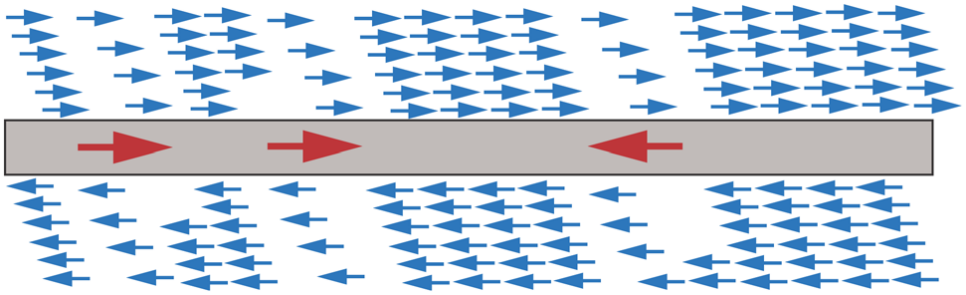
Tiling strategy. The genome was divided into coding and non-coding regions, and the two region types were probed with different densities. The grey bar represent the genome, red arrows represent genes and the blue arrows represent probes. The numbers of probes are not realistic here (see Table S1 for density details).

A limited number of features on the arrays often prohibits a high density tiling strategy from covering the entire genome evenly. As these chips have a 280,000 feature size limit, the decision to split the genomes into two categories was taken; coding and non-coding. All regions annotated with an Open Reading Frame (ORF) having a known function on either strand were defined as coding regions, ORFs separated by less than 25 nucleotides were concatenated. The remaining regions were defined as intergenic (Figure 1). This process of dividing the genome into two categories does not introduce any bias to the applied analysis method, and is solely used for the purpose of probe design as the feature number is limited. For the genomes used in this design, the intergenic regions make up about 10 percent of the *E.coli* and 20 percent of the *N. meningitidis* genome. The terms “coding” and “non-coding” are used here *only* to describe the two categories defined during the design phase.

As *E. coli* and *N. meningitidis* differ in genome-sizes as well as the percentages of non-coding versus protein-coding regions, the probe densities in the coding and non-coding parts in the two genomes were set independently. This density trade-off was dictated by the percentage of coding and non-coding regions along with the total feature number available. The coding regions were covered by 19 and 32 probes per gene in *E. coli* and *N. meningitidis*, respectively. The probe density parameter details can be found in Table S1.

Several probe selection programs are available today, such as OligoArray 2.0 [19], CommOligo [20], OligoWiz 2.0 [21], [22] and a web tool from the Gerstein lab (http://tiling.gerstein.org) [23]. OligoArray 2.0 from 2005 was designed for automated selection of short oligonucleotide probe sequences, it requires BLAST and uses MFOLD [24] for thermodynamic secondary structure and probe specificity predictions. CommOligo, accompanied by the Comm Oligo Parameter Estimator, on the other hand addresses whole genome array design or probe design from highly homologous sequences. OligoWiz 2.0, which is applied here, is an oligonucleotide selection software with several user defined parameters; ΔT_m_, homology, low-complexity, position and “GATC” only, probe spacing and a maximum and a minimum probe number per sequence. The two algorithms from Bertone *et al.* [23] that form the Gerstein lab web tool concentrate on eukaryotic genome tiling, hence detection of similar probes or sub-sequences between probes is their main focus. Their work emphasise the value of a tiling strategy which optimises the probe affinities rather than a uniform tiling solution, as long as the obtained coverage is sufficient to answer the biological question asked.

As the target organisms here are bacteria, the large-scale eukaryotic similarity problems are excluded (i.e. the Gerstein lab web tool solution) and since the homology problems in bacteria are relatively small, the need for the CommOligo special functionality relating to probe designs for highly homologous sequences is not as critical as for higher species/organisms. To make the initial oligo selection, OligoWiz 2.0 was chosen on the basis of functionality, and the implemented selection algorithms were well suited to the tiling design in these specific projects. Major factors contributing to the selection of OligoWiz 2.0 were the ability to adjust the score parameters to fit the selective tiling design and to apply different probe densities for known ORFs and intergenic regions. In addition, OligoWiz 2.0 is more compatible, since it can be run without the position score-filter, since every part of each probed region is equally important in terms of the detection of novel transcripts. Some recent methods for probe selection are discussed in the “Conclusion and method remarks” section at the end.

After the divison into coding and non-coding regions, the initial selection of probes was made using OligoWiz 2.0 [21], [22]. From the resulting set of all possible probes, a subset was chosen by setting the selection parameters in OligoWiz 2.0 (see Tables S2 and S3). When choosing a small minimum inter-probe distance (≪probe-length) for the intergenic regions a “selective tiling” is achieved, i.e. high density, but with high quality probes only (see Table S1 for maximum probe density.) Repeat regions were not removed prior to the probe selection, but were avoided by the combination of OligoWiz 2.0 criteria followed by subsequent probe selection scripts. The main function of these scripts was to remove duplicates, see “probe-uniqueness” below. On the actual array no genomically adjacent probes were closely located on the chip, in order to minimize errors from spatial effects.

To ensure sufficient coverage of both strands, every probe on the array has a complementary probe (if unique) covering the opposite strand. This complementary design also enables all probes to be hybridized with DNA or RNA from both strands. One should keep in mind that hybridization to total DNA can give good probe-quality measurements, which is a useful mean for experimental probe-quality assessment [10]. To achieve this design, OligoWiz 2.0 was applied on one strand and then all probes were complemented to cover the reverse strand. Each complement probe was assigned the same score as its origin. Test-runs with OligoWiz 2.0 proved this approach reliable compared to applying OligoWiz 2.0 on both strands. The complementary probes were then checked for uniqueness (see below), and removed if non-unique (exemplified by the removal of 166 out of 273.414 probes from the original *E. coli* design).

The optimal melting temperature was estimated by OligoWiz 2.0. All regions were considered equally important, as the goal was to map the entire transcriptome. Therefore, the OligoWiz 2.0 position score was left unused. For future designs, variable probe length design (24–26 mers) might be considered in order to achieve a more uniform melting temperature distribution for all probes [25].

Cross-hybridization occurs when a piece of cDNA in the sample binds with, and hence add signal to, a probe that is not 100% complementary. This results in false positives that are almost impossible to identify and remove. This is considered to be a critical problem in array designs [26]. Therefore, the cross-hybridization threshold was the most heavily weighted score. The related sequence–complexity score was also set reasonably high to further decrease the risk of cross-hybridization, see Table S2. One major drawback regarding the probes selected by OligoWiz 2.0 is that the program is able to select identical probes from two different input sequences. The program can thus report two good probes while actually choosing two identical probe sequences. Similar probes on the chip therefore make it impossible to map the actual transcript back to the genome. To avoid this problem of non-unique probes, a computer program removing duplicates from the OligoWiz 2.0 output-files was written and applied (available upon request). The script uses a hash-table with all 13 nucleotide sub-sequences of all probes as keys, if similar keys are detected, all non-overlapping probes with this sub-sequence are removed. This allows a maximum similar continuous stretch of 12 nts. The removal is followed by a control of the regions from which the probes have been taken away. If the removal strongly affects the coverage, another probe with a lower OligoWiz 2.0 score is selected, from the set of all possible probe-sequences generated, to ensure sufficient probe coverage.

A quality assessment of the sample preparation, the hybridization-process and the intensity measurements can be obtained by using control-probes [27]. Control probes are sequences foreign to the target genome designed to assess cross-hybridization and background noise. There are several commercial sets of control probes made to measure the hybridization quality, as well as the RNA sample preparation, labelling and fragmentation process [28]. An improvement of the data quality measurement is sought here by the inclusion of multiple control sets in combination with multiple copies of each control probe. By distributing six copies of these control probes (seven including the hybridization controls, see Figure S1) around the arrays, more measurements can be taken to improve the quality control process. This control probe distribution is used particularly to assess chip-area specific hybridization artefacts. In total there are 4566 control probes distributed over seven separate patches on the chip, see Figure S1 and Table S4 for details. The standard controls used on these arrays are the Affymetrix hybridization control-set, the Affymetrix prokaryotic spike-in set (poly-A) for assessment of the sample preparation and labelling process and the HXB2-yeast spike-set (all three sets described in [28]). Additionally there is a custom made control probe-set consisting of 50 probes having a di-nucleotide composition similar to the *E.coli* specific probes. These custom probes were generated by computing all di-nucleotide frequencies for the target genome probe sequences. Then a probabilistic algorithm producing 25-mers with similar di-nucleotide composition to the target specific probes was implemented. The algorithm outputs the N first probes that differ on at least seven out of 25 nucleotide positions when compared to every *E. coli* specific probe.

The design method presented here was originally made for relatively small genomes (4×10^6^). However, the design is easily adapted and scaled up to larger genomes. The target genome size and the feature number available, combined with the biological question asked, will decide whether a tiling approach with equidistantly distributed probes of the entire genome is possible or not. If this approach is considered, Gräf *et al.* [29] as well as Schliep *et al.* [30] recently presented more suitable methods for equidistant probing. The method presented here is on the other hand an elegant alternative for non-equidistant tiling designs. We believe that the division of the target genome into “high” and “less high” interest regions is trivial after the biological question has been stated. OligoWiz 2.0, or another well suited oligo selection tool depending on the biological question (see “Method remarks” section and [31]), should then be applied to design probes suitable for the feature number available and the resolution needed in the genomic region of interest. A probe selection as described here will then select the set of best unique probes for the final design. The control of uniqueness described here can be exchanged for a suffix array approach [32], if the hash-based method raises memory-limit problems. Also, if splice-variant related questions are raised, probes must be designed with probe sequences that represent both the end of exon_A_ and the start of exon_B_, as used by Skotheim *et al.* [33]. The control probe design method, including the random negative controls, is well suited to any genome or array size.

### Data normalisation

**Figure 2:**
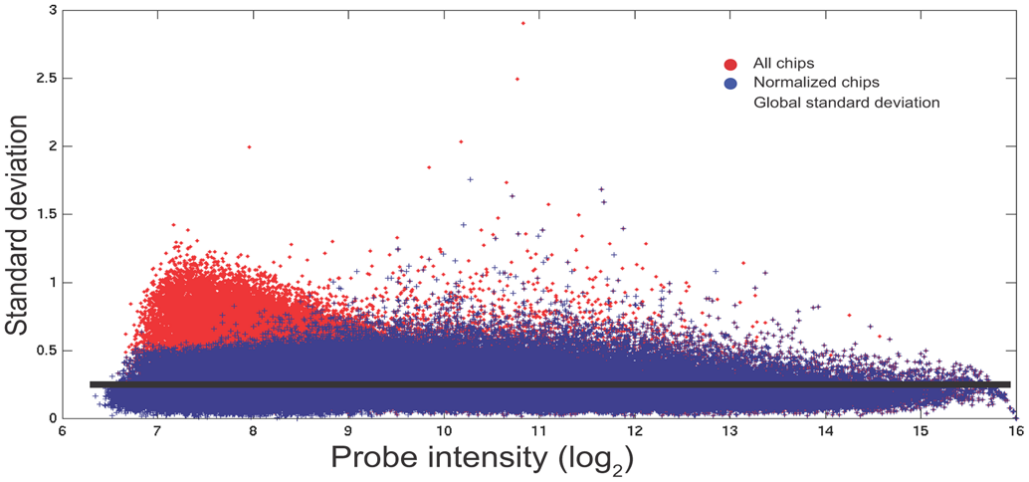
Standard deviation versus intensity for all probe sets. Plotting standard deviation versus intensity for all probes across the 5 arrays (red circles) allowed a mean level of interest to be calculated for the standard deviation. This was considered as a global measure of the standard deviation (σ_g_) between probes in the set of 5 arrays. All extreme outliers were removed (see text for details) and the result from this filtering is shown by blue circles.

There are a number of accepted normalization techniques that can be applied to microarray data, with varying levels of complexity and transparency. In many experiments, normalisation procedures have proved extremely advantageous; but, as discussed elsewhere [34], in the cases of relatively small genomes such as that of *E. coli* (∼4.6 Mbp) and *N. meningitidis* (∼2.3 Mbp) the benefits are usually minimal and the application of complex sequence based normalisation routines can in fact confound otherwise clean data (See File S1 for full discussion). It follows, therefore, that it is preferable to minimise normalisation solely to the removal of significant outliers from the data. Ideally, data from multiple arrays show a variance between the log_2_ intensities of a single probe-set, which is independent of the mean log_2_ intensity for the given probes for all but the extremes of the data. Plotting the standard deviation versus the intensity for all probe-sets after aligning the data by the mean values of all chips (red circles in Figure 2) allowed a mean level to be calculated for the standard deviation. This was considered as a global measure of the standard deviation (σ_g_) between probes in the set of 5 chips (see Figure 2). The global standard deviation was then used to process the data set, by removing the worst-case outliers from the data sets. Here, exactly 46,321 out of 2,733,980 data points were removed from the MNNG experiment. Outlier detection was performed by sorting the five different array signal values from each probe into ascending order and taking the mean of the middle three points as the central value. If either of the remaining probes was found to be more than three global standard deviations (3σ_g_) from the central mean value it was considered to be an outlier with >99% certainty and was therefore discarded. In all other cases, the probe values were retained. The result of this probe outlier filtering is shown as blue circles (Figure 2). This was done before a comparison of relative expression levels was performed on the data.

**Figure 3:**
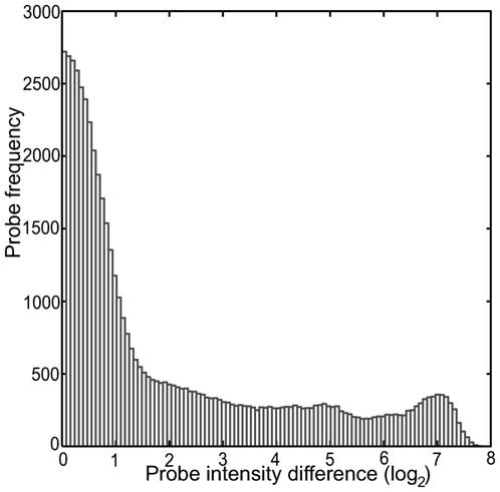
Probewise difference distribution between normalisation methods. Distribution of differences between our normalised data and the gcRMA normalised data is shown. Y-axis represent probe frequencies and the X-axis the absolute value of the difference (log_2_).

Given that adjacent probes within a single gene may differ in signal with a standard deviation >1 (on a log2 scale) [35] we have the option to create a very conservative dataset by selectively removing probes using the results of the gcRMA algorithm [11] run on the original raw dataset, in comparison to the dataset returned by the normalization procedure described above. As the original gcRMA algorithm (version 1.0) uses mismatch (MM) probes we applied gcRMA 2.0 (http://rss.acs.unt.edu/Rdoc/library/gcrma/doc/gcrma2.0.pdf). Our custom designed random negative control probes where used in the “bg.adjust.gcrma()” method call, that adjusts for background signals, instead of MM probes. Approximately 10% of all probes (28.594 out of 273.398 in the referred MNNG experiment) can subsequently be discarded where the difference between the gcRMA results and normalized data exceeded the set threshold. The threshold difference level was defined on the basis of the distribution of mean differences between the control and stressed data sets (Figure 3). At extreme difference values, >6 (log_2_), there is clearly a secondary peak in the distribution, contributed by data points, which are in strong disagreement with the gcRMA algorithm. In order to minimize data adjustment, while removing the points with strongest disagreement, the threshold difference was set in the minimum region of the distribution between primary and secondary peak.

**Figure 4:**
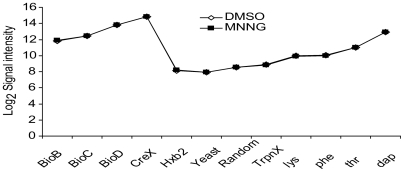
Reference and MNNG treated *E. coli* control probeset average intensities. Average signal intensities for all control probes in reference (Dimethyl Sulfoxide Reductase (DMSO) added only) and treated (N-methyl-N'-nitro-N-nitrosoguanidine (MNNG)) *E. coli*. It is easily seen that the lines overlap very well (sometimes one is hidden by the other), and hence the two experiments can easily be compared with only a minor baseline shift.

**Figure 5:**
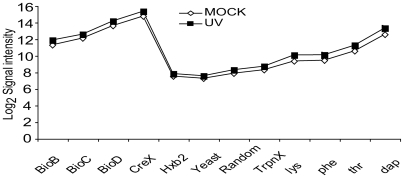
Reference and UV treated control probeset average intensities. Average intensity for all control probes in reference (Mock) and treated (UV irradiated) *E. coli*. Note the consistent difference on all spiked in genes.

**Figure 6:**
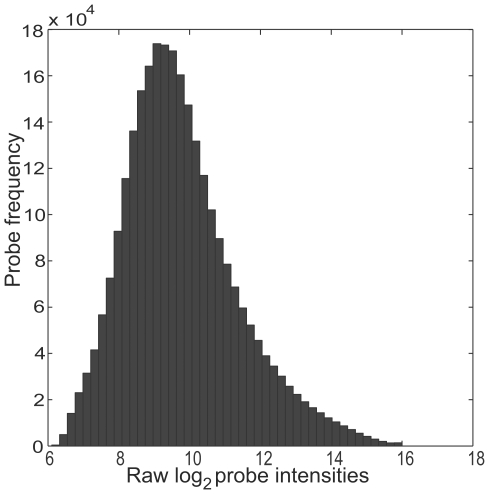
Raw data signal intensity distribution. Signal intensity distribution of all probes for reference (DMSO) and treated (MNNG treated) *E. coli* before data processing. Log_2_ signal intensities on the X-axis and probe frequencies on the Y-axis.

As previously stated, the large number of control probes assured good assessment of the labelling and hybridization process, respectively. The average signal intensity values of all the spike-in probes for two experiments with a reference and a treated dataset are shown in Figures 4 and 5. The intensities of non-specific probes (HXB2-yeast-, random- and *trpnX*-probes) give an estimate of the level of cross-hybridisation and background noise. An interesting observation is that HXB2-yeast spike set has slightly lower average signals than the custom-made experiment specific control probes, indicating that custom-made, genome specific, negative controls might be better for background signal estimation than these standard spike-sets. The, custom controls show a higher and probably more correct background signal intensity level than the standard sets. The background level was defined as the level at which low level transcription becomes indistinguishable from other background signals. Since low-level transcription predominates along the total length of the genome, this low-level intensity is defined by the peak of a histogram of probe intensities (Figure 6). Below this level it is impossible to separate error from transcription levels. Therefore the background level was set to a log_2_ intensity level of 9.0 for the *E. coli* arrays, which is a slightly higher level than the intensities of the custom negative control probes (Figure 4 and 5). All signals below the background noise level are considered as uncertain since they might be a result of noise and/or cross-hybridisation.

Scaling of experimental data should be performed when comparing two datasets where a consistent difference can be detected between control probes designed to give equal signals at a range of different intensities. Here, the average difference showed little variation between probes at differing intensities and therefore the difference was applied as the baseline shift of the reference dataset (Figure 4 and 5).

### Probe-specific effects and estimation of the minimum length of a trustworthy signal

One important question regarding tiling arrays is how long a region is needed to be for its signal to be considered a true signal? A short stretch of the genome with unusual base-composition might result in probes with a very high or very low binding affinity [11]. Probes having low binding affinity might give rise to false negatives, while the ones with high affinity can produce false positives *only* when looking at the expression levels, and false negatives *only* when considering differentially expressed regions. These possible high or low affinity probes could be removed by the application of the gcRMA [11] based method described previously to the raw data. This decreased the number of probes that potentially have biased signal intensities due to highly diverging probe-affinities, although the design process tries to avoid such differences. In the case of differentially expressed regions, probe-artefacts should be equivalent in both conditions and hence regions detected as differentially expressed should be trusted, although there might be uncertainties connected to the absolute signal intensity values, due to the probe affinity problem.

**Figure 7:**
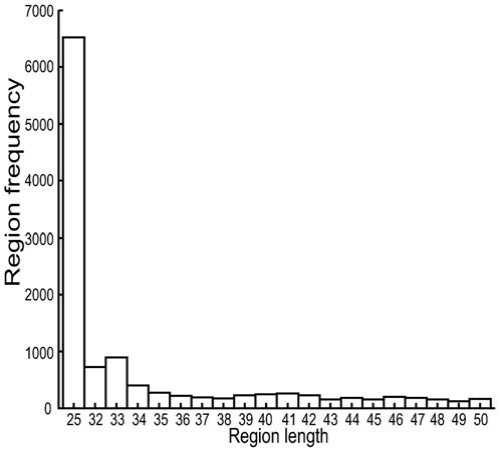
Distribution of short similarly expressed regions. Distribution plot of all similarly expressed regions (< = 50 nts in length) in the DMSO and the MNNG dataset

Similarly expressed regions and regions detected as present are a different matter. First one must consider the very high probe density, which inevitably will give rise to probes with diverging affinities, even though this has been striven against in the design and normalization process. Thus, with strict selection criteria, regions that are transcribed *in vivo* as long stretches of RNA might appear to be divided into several shorter stretches by the presence of low-affinity probes. On the other hand, short stretches appearing to be expressed in both conditions might be a result of probe artefacts, indicating that they might represent false positives. In addition to this, cDNA production and RNA degradation may, to some degree, represent certain sources of errors. This would likely be due to shortened or missing cDNA pieces from the sample, generating false negatives. Bearing in mind the above observations, differentially expressed regions with a length of only one probe (25 nts) will be considered significant in this study. To define a minimum length threshold for regions detected as present, or similarly expressed, the length distribution of the expressed regions (≤50 nts) with a signal above the background level were plotted in a histogram (Figure 7). A cut-off of minimum 36 nts was set based on this distribution plot combined with the criterion of a separation of the two adjacent probes by at least 10 nts to ensure specific binding of the cDNA to both probes. The minimum spacing criteria of 10 nts is based on the Roche NimbleGen design guide [36]). This exclusion will inevitably exclude true positives, but still it will remove far more false positives and in the end increase the overall data-quality.

### Analysis methods

The era of tiling arrays is fairly new [6] and there is not yet one preferred, established and thoroughly tested data analysis method. One problem is that most commercial and free-ware analysis tools are made solely for traditional gene arrays and are therefore not designed to handle the tiling strategy. Therefore the researcher has to create new functions to sub-optimal programs already available, or develop new data analysis tools to fit their specific need.

The percentage of transcribed DNA compared to total DNA is unknown with regards to the bacterial genomes considered in this paper, but is believed to be significantly higher than the percentage annotated today (based on previous tiling projects [37]–[42]). Nonetheless, tiling arrays are supposed to show far fewer high-intensity signals than normal for gene-targeting arrays probing only coding regions. When intergenic regions are probed there are no defined areas in which to look for signals, hence new considerations and adjustments have to be made.

As the goal of this project was divided into transcriptome mapping and detection of differentially as well as similarly expressed genes and transcripts, including novel short transcripts, different analysis methods needed to be developed. First, an annotation guided approach was applied in order to investigate similarly and differentially expressed annotated genes between reference and treated cells. Then, a novel and more complex sliding/expanding window approach, independent of previous annotations, was developed to segment the data and give a comparative analysis of the tiling-results. This approach also allowed transcriptome mapping independent of the comparison between reference and stress datasets.

#### Annotation based method

In the annotation guided approach all probe signals for each condition of an annotated gene were collected into two groups X*_n_* and Y*_m_*. X*_i_* is probe *i* of a total of *n* probes probing the reference sample, while Y_j_ is probe *j* of a total of *m* probes probing the treated sample. As a result of the probe-by-probe normalization method, *n* and *m* are not necessarily equal. A two-tailed unpaired t-test was applied to compare the means of the signal values X*_n_* and Y*_m_*. A p-value of 0.05 was chosen as the threshold for rejection of the null hypothesis that the mean values of the two probe sets originate from the same distribution. This threshold equates to a 0.95 confidence of a differential expression between reference and treated data sets. Probe sets conforming to this condition were logged as candidates for differentially expressed genes. Subsequently the absolute average signal intensity difference (fold-change) between all X*_n_* and Y*_m_* probes was calculated. Genes having a probability > = 0.95 for differential regulation combined with an absolute fold-change > = 0.5 were finally considered as differentially expressed. In cases where the average of X*_n_* or Y*_m_* was below background signal, this average was adjusted to be equal to the background signal before the fold-change calculation was made. This excluded the possibility of false positives in difference calculations occurring due to the presence of erroneous low signals. Although it may be argued that the use of a t-test is suboptimal in cases where many probes are present in an annotated region, the subsequent application of the fold change rule ensures that regions defined as differentially expressed are valid. Meanwhile, when attempting to distinguish differential expression in the shortest fragments, which is our primary interest, application of the t-test as the first rule is the optimal solution.

The p-value returned by each t-test was recorded and subjected to a Bonferroni multiple-testing correction. In practice, these p-values were so small (≪0.05) that the entire genelist measured as differentially expressed all pass the Bonferroni test. Similar results were shown for the t-tests applied to the top two-hundred regions identified by the sliding window method (below).

Genes where the average of X*_n_*> = background and the average of Y*_m_*> = background and the probability of differential expression or the fold change was below either threshold value were considered similarly expressed. We are aware that a more correct term would be non-significantly differentially expressed but for simplicity similarly expressed is used. Genes having either the average X*_n_* or Y*_m_* below the background level were excluded, as the true signal value is uncertain. Inclusion could lead to false positives, while exclusion gives possible false negatives. The false negatives might be further investigated by looking at the dataset from the plain transcriptome-mapping data (see present/absent regions further below). The background adjustment is, as for the differentially expressed genes, adjusted for the “worst-case” scenario.

#### Sliding and expanding window method

The normalized data, i.e. after removal of datapoints defined as outliers compared to the gcRMA-normalized data, was sorted according to strand and genomic position.

**Figure 8:**
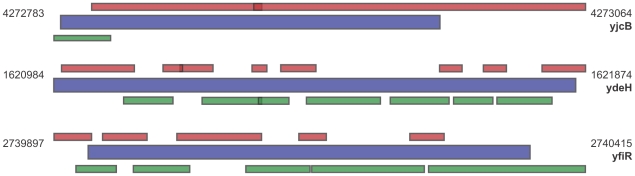
Genes reported as differentially and similarly expressed. A visualisation graph of how several regions can cover one single gene. The blue bars represent genes, differentially expressed regions are represented by the brown horizontal bars above the genes and similarly expressed regions are represented by the green bars below. The numbers indicate genomic start and stop coordinates.

A sliding and expanding window algorithm was then applied to run along the probes in order to perform calculations on window-sizes of one, three and five probes, for each consecutive probe. For every probe along the genome, a score (0 or 1) was computed for each of the three window sizes. First, an unpaired t-test was applied to calculate the probability of differential regulation between the reference and the stressed samples within the window. Second, the absolute difference of the average signal intensities (fold-change) of all the signals inside the window was computed. Third, the probability and the fold-change were used to define a boolean set of zeroes or ones for differential expression in each window at each probe-position where a 1 indicate that the window has a probability > = 0.95 for being differentially regulated, combined with a fold-change > = 0.5 (log_2_ value). On the other hand a 0 indicates that the probability and/or the fold-change criteria of differential expression are not met. Furthermore, no window could include regulation in both directions, if the window received a score of 1. This sliding and expanding window algorithm resulted in two large score matrices, one for each strand (example in Table 1). A selection algorithm was then applied on these score-matrices. This algorithm searches through the matrices sequentially and selects regions that are differentially regulated. Differentially regulated regions are identified by locating rows in the matrices where all window sizes (1 through 5) had a score of 1 and continues if the next row in the matrix is equal to one of the following [1 X X] or [0 1 1], where X can be either 0 or 1. If a single matrix row of [0 0 1] is located between two rows fulfilling either of the mentioned criteria, this row is also included in the differentially expressed region. In addition, the regulation has to be uniform (either up or down) on all the probes inside a detected region. For all regions detected, the overall t-test score and fold-change value was computed. The final step of the region-selecting algorithm was to annotate all the detected regions. This was performed by searching for genes overlapping on the same or the opposite strand. If no such overlap was found, the distance to the closest upstream and downstream genes were calculated. For all regions not detected as differentially regulated, another algorithm was applied that located all similarly expressed regions, i.e. regions where both datasets had a signal average >background level but with t-test probability and/or the fold-change level below the threshold of a differentially regulated region, (0.95 and 0.5 respectively). Finally, all the similarly expressed regions were annotated as described earlier. As this method is independent of previous annotations, genes might be reported as partly similarly and partly differentially expressed. Also, there might be some overlap (<25 nt) between regions being differentially and similarly expressed due to the algorithm selection criteria and the overlapping probes (Figure 8).

**Table 1:** Strand-wise score matrix from the Sliding window algorithm.

Probe start	Probe end	Window-size 1	Window-size 3	Window-size 5
49	74	0	1	1
*57*	*82*	*1*	*1*	*1*
*64*	*89*	*1*	*1*	*1*
*72*	*97*	*0*	*0*	*1*
*79*	*104*	*1*	*1*	*1*
*86*	*111*	*1*	*0*	*1*
*94*	*119*	*0*	*0*	*1*
*102*	*127*	*1*	*1*	*0*
*110*	*135*	*1*	*0*	*1*
*119*	*144*	*1*	*1*	*1*
*129*	*154*	*0*	*1*	*1*
*137*	*162*	*1*	*1*	*0*
145	170	0	1	0
152	177	1	0	0
165	190	1	1	0
195	220	0	1	0
229	254	1	1	0

A strand-wise score matrix generated by the sliding window algorithm. The example is fictional and illustrates different examples of how the algorithm expands a differentially expressed region. The region from 57 to 162 (*italics*) will be detected as differentially expressed, while the rest are non-differentially expressed regions.

#### Transcriptome mapping

An expressed region is a continuous stretch of probes that on average show a signal intensity value above the background noise level. All regions not detected as expressed (scored present) were reported as absent, i.e. missing. This present and absent calculation was done for the samples independently prior to the annotation procedure. Regions excluded by the applied algorithms for the selection of differentially and similarly expressed regions within the confines of the methods described above, can be investigated by comparing the present and absent data for the samples.

### Normalisation method comparisons

The issue of normalization is critical in microarray experiments, since the data quality can be highly dependent upon the chosen algorithm. In the case of these custom arrays designed using the OligoWiz 2.0 probe selection program, a visual inspection of the data after application of the gcRMA normalization method [11] indicated data quality degradation. In order to quantify this impression we extracted the 87637 probe values from regions that are annotated and therefore expected to be consistently expressed. The strategy chosen was to use the mean value of all probes within a single similarly-expressed region in order to define the transcription level within this region. This led to the possibility to calculate the deviation – or sequence-dependent bias – of each individual probe from the mean transcription level. The measured biases were, as would reasonably be expected, normally distributed around zero. The quality of any normalizing algorithm was then easily defined by its influence on the normal distribution. A worthwhile normalization method would result in a reduction of the observed variance, while any increase in the variance would imply no improvement to the data quality, thus telling us that the chosen method is wrong for the dataset. Comparison of the variance between probes normalized by our method and the equivalent gcRMA normalized probes showed a variance of 1.17 and 6.84, respectively Therefore, in this case, application of the gcRMA method *severely* degrades the data quality. This in itself is intriguing and leads us to conclude that the design setup and the application of OligoWiz 2.0 (choosing uniform T_m_ values and GC-content) for probe selection defines a probe set which is incompatible with the gcRMA algorithm. The relative concentration of non-coding compared to coding region probes on our chips will also work against the gcRMA algorithm. Additionally the substitution of MM probes with random control probes, presumably having higher intensities than regular MM probes, will confuse the gcRMA algorithm. The decision was therefore taken not to apply any further normalisation to the data. (See discussion in File S1)

**Figure 9:**
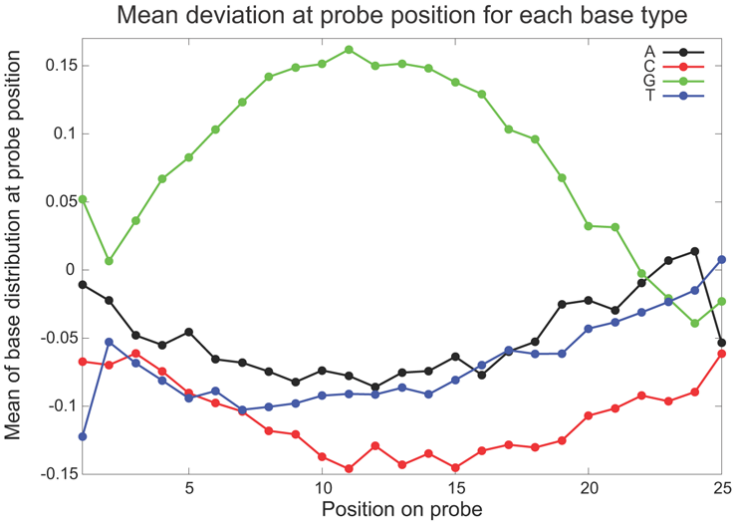
Probe nucleotide composition bias. Mean bias for each nucleotide type at each position along the probe for all probes within known annotated regions of the genome, illustrating the basis of the sequence dependence of individual probe biases.

As a further exercise in understanding the sequence dependence of the bias, we compiled our data into histograms of the bias for each nucleotide type at each position along the probe (see Figure S3) and used this to generate a graph of the mean bias for each nucleotide at each position along the probe (Figure 9), which would act as the basis for any sequence dependent bias estimate. This is markedly different to the curve shown by Wu *et al.* and in their discussion of gcRMA [11], further confirming the incompatibility of our probe set with the gcRMA normalisation. Taking this even one step further and applying a generalized linear model (GLM), incorporating single nucleotide positions to the measured biases (using the SAS statistical package) we subsequently produced a set of additive coefficients for individual nucleotide positions (see Figure S2 and Table S5) with which sequence specific probe bias corrections could be made to the data set. Application of this sequence based correction show that a reduction in bias variance from 1.17 to 0.95 was attainable; thus implying that some sequence based normalization is achievable. Due to the time constraints imposed by related biological experiments that were necessary in order to confirm stress responses measured using this microarray data, this fine-tuning normalisation was not applied to the published data sets; however we include the outline of what is possible for the sake of completeness.

To investigate whether our probesets are compatible with standard normalization methods, gcRMA regular RMA and VSN [43] were applied to the data, and a variation comparison study was conducted. Details of these tests are in File S1, but the conclusion showed quite clearly that all three methods made the signal-to-noise ratio worse than unnormalized data. Thus we are vindicated in our choice not to apply standard methods.

## Results and Discussion

Different genomes have different nucleotide-compositions, and one should always ensure that regions of special interest on the target genome have a sufficient coverage of probes. This is to ensure that no important genomic region goes un-probed due to some nucleotide composition abnormality.

Here we present a novel method that enables detection novel short (<60 nts) intergenic transcripts by custom made tiling arrays. To ensure sufficient intergenic coverage, overlapping tiling of probes was used in all intergenic regions (as far as the probe quality thresholds allowed). For the *E. coli* genome, a feature number of 386,000 is needed for a complete non-overlapping tiling. Since the array feature number (∼280,000) was below 386,000 non-equidistant probing was applied. This probing strategy, which is considered dense, gives a very high intergenic coverage (up to 7 nt resolution), On the other hand, it gives sufficient coverage within regions of known genes. This probe density trade-off is balanced between the feature number and the biological questions asked. With our strict definition of coding and non-coding regions (see above) the applied design solution was considered optimal in terms of the biological aims. During the analysis of the arrays we have reconsidered this and would recommend equidistant coverage of coding regions combined with overlapping tiling of regions of special interest, if the total feature number does not allow dense coverage of the entire genome. In our existing case, the equal probe coverage of each known ORF implies equal data material for each gene to base the statistical analysis on and potentially enables the discovery of more individual gene features [31]. In the suggested case, probes should be tiled as densely as the feature number and the probe quality prediction allows.

Furthermore, by randomly distributing the control probes rather than grouping them in blocks as done here, one might obtain even better assessments of spatial bias. In the end it is the biological question underlying the design that decides where probes are of most efficient use. We still consider “selective tiling” better than a plain equidistant tiling approach, as high or low affinity probes would have to be heavily adjusted or thrown away during background predictions or normalisation procedures anyway. Additionally, a somewhat surprising increased transcription detected, and biologically validated, in regions opposite to some known genes indicate that, if the feature number allows, such regions should be prioritized with denser coverage

One may also think of experimenting with even more similar custom made control probes to find the “optimal similarity” when assessing background noise.

It should be noted that although OligoWiz 2.0 strives to obtain uniform probe affinities. Therefore, probe designers should be observant when designing probes for genomes with GC-content far higher or lower than 50%, as OligoWiz 2.0 has no GC-specific scoring filter. The GC-content is closely related to the T_m_ score and OligoWiz 2.0 would still select probes with uniform binding affinities but the optimal hybridisation temperature would be different and there are possibilities of a decrease or increase of cross-hybridisation due to the GC-content.

Since the actual array design several novel design algorithms and software have been introduced to the research community and are elegantly reviewed and compared in a recent study by Lemoine *et al.* [31]. Lemoine *et al.* show that OligoWiz 2.0 stand out as one of the best choices, as long as the studied organism is found in the OligoWiz 2.0 database. Of the competitors, CommOligo [20] could be considered if the target organism has a non-regular GC content or higher organisms with low-complexity regions. And ArrayOligoSelector [44] or OligoTiler (http://tiling.gersteinlab.org) should be considered when designing tiling arrays with feature numbers sufficient to provide equidistantly spacing of probes combined with sufficient coverage to answer the biological question asked.

Even though the tiling array technology has been around for several years now there is still no “all-in-one” programs and little “how-to” information are available. A few programs/algorithms have been developed for creating oligonucleotide tiling arrays [23], [45], [46] but none of these have the multi functionality that a chip-designer ideally would hope for. Also, as the interest in specific bacteria differs, one design algorithm might not give good results for two different species without modification.

The annotation based analysis method is a simple and straightforward method for the analysis of the coding parts of tiling experiments. But one should be aware that this method relies on *known* annotations. The sliding window approach, on the other hand, is novel *but* independent of previous annotations. This method is somewhat more complicated and time consuming. The array design, normalisation and data analysis methods presented here have produced a mass of biologically relevant results (manuscript in progress). This shows that the strategy from this work can be implemented on bacterial genomes, and on eukaryotic genomes after applying the minor changes suggested.

### Additional information

The array definition and the datasets from the *E. coli* study has been submitted to the Gene Expression Omnibus [47] with accession number GSE 13829 and 13830 (data) and GPL 7714 (array). All computer programs made by the authors have been written in Python and MATLAB and can be obtained on request.

## Supporting Information

Figure S1 Control probe distribution
(0.09 MB PDF)

Figure S2 Nucleotide position bias
(0.39 MB PDF)

Figure S3 Nucleotide bias histograms
(0.87 MB PDF)

Table S1 Probe density parameter overview
(0.05 MB PDF)

Table S2 Initial OligoWiz 2.0 parameter settings
(0.05 MB PDF)

Table S3 Final OligoWiz 2.0 score-weight parameters
(0.05 MB PDF)

Table S4 Overview of the control probes
(0.07 MB PDF)

Table S5 Additive coefficients for sequence specific bias adjustments
(0.07 MB PDF)

File S1 Supplementary file with a thorough discussion about the quality assessments done on the presented normalisation method and comparisons to gcRMA, RMA and VSN.
(3.30 MB PDF)
