# Supplementary material for: Custom Design and Analysis of High-Density Oligonucleotide Bacterial Tiling Microarrays
Source: PLoS One. 2009 Jun 17;4(6):e5943. doi: 10.1371/journal.pone.0005943 (PMC2691959; doi:10.1371/journal.pone.0005943)
Supplement: Figure S1 — Control probe distribution (0.09 MB PDF) [file pone.0005943.s001.pdf]

Figure S1. Control probe distribution

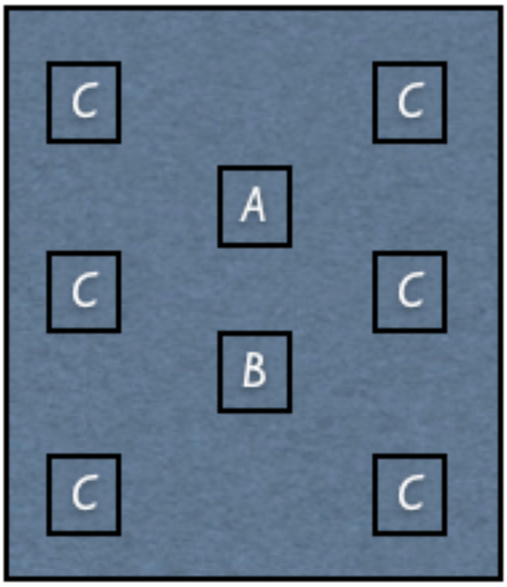

Distribution the control probes on the microarray. Region *A* holds internal Affymetrix production controls, region *B* holds PM and MM probes for the standard hybridization controls and all regions marked *C* holds one copy of the 50 random probes, the standard Affymetrix hybridization probes and the HXB2-yeast probe-set. Better coverage when considering chip-area specific quality can clearly be seen when compared to using only control section *A* and *B* only. For future work randomized placement of extra control probes should be considered as an alternative to this grid-like placement.
