# Supplementary material for: Custom Design and Analysis of High-Density Oligonucleotide Bacterial Tiling Microarrays
Source: PLoS One. 2009 Jun 17;4(6):e5943. doi: 10.1371/journal.pone.0005943 (PMC2691959; doi:10.1371/journal.pone.0005943)
Supplement: Figure S2 — Nucleotide position bias (0.39 MB PDF) [file pone.0005943.s002.pdf]

Figure S2. Nucleotide position bias

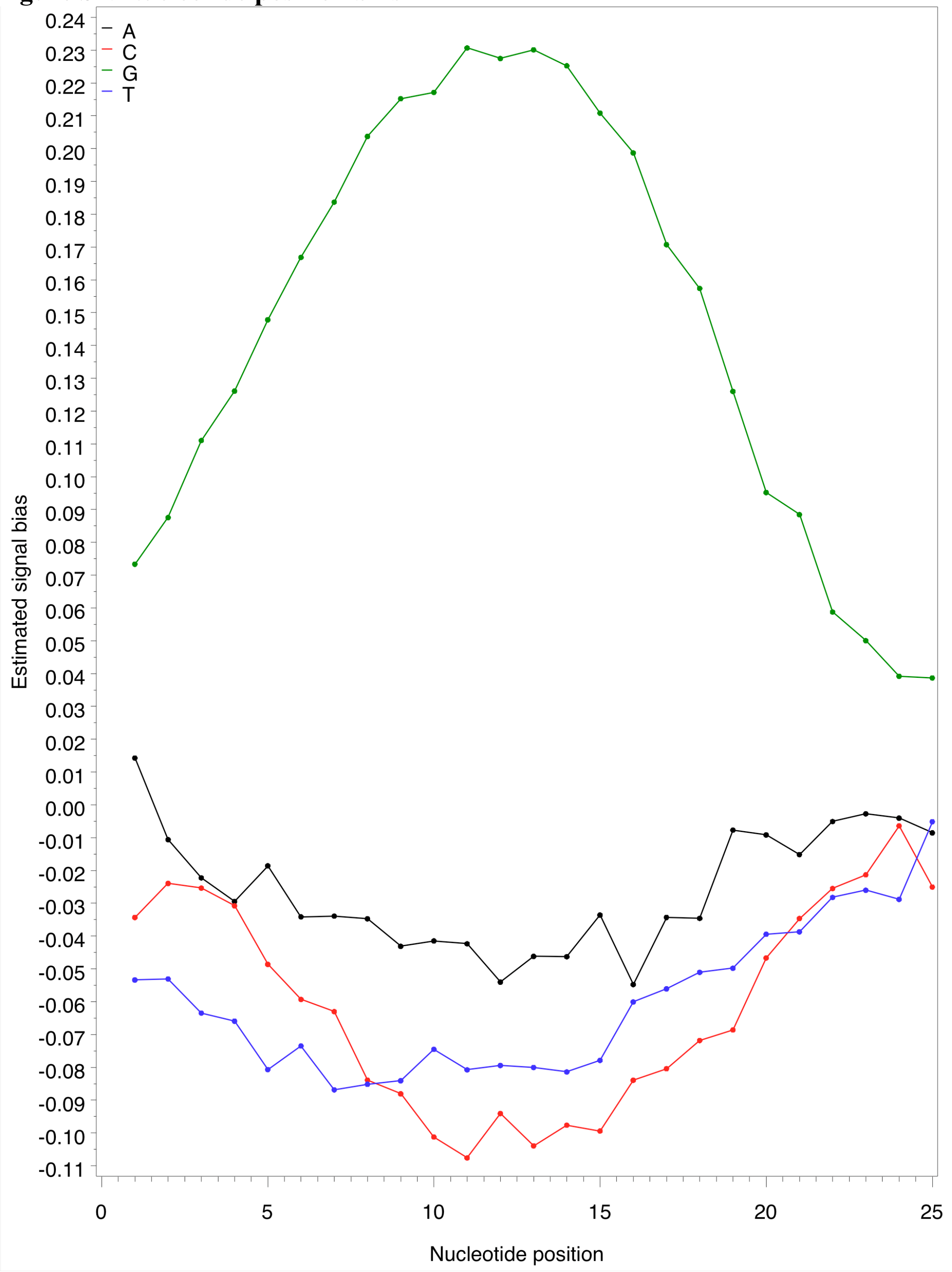

Figure showing additive coefficients for each probe position, by means of which a proportion of the bias for a given probe can be estimated. Application of these normalisation coefficients to the data showed a global reduction in bias variance as discussed in the article, proving that it can function as a normalization routine.
