# Supplementary material for: Custom Design and Analysis of High-Density Oligonucleotide Bacterial Tiling Microarrays
Source: PLoS One. 2009 Jun 17;4(6):e5943. doi: 10.1371/journal.pone.0005943 (PMC2691959; doi:10.1371/journal.pone.0005943)
Supplement: Figure S3 — Nucleotide bias histograms (0.87 MB PDF) [file pone.0005943.s003.pdf]

Figure S3. Nucleotide bias histograms

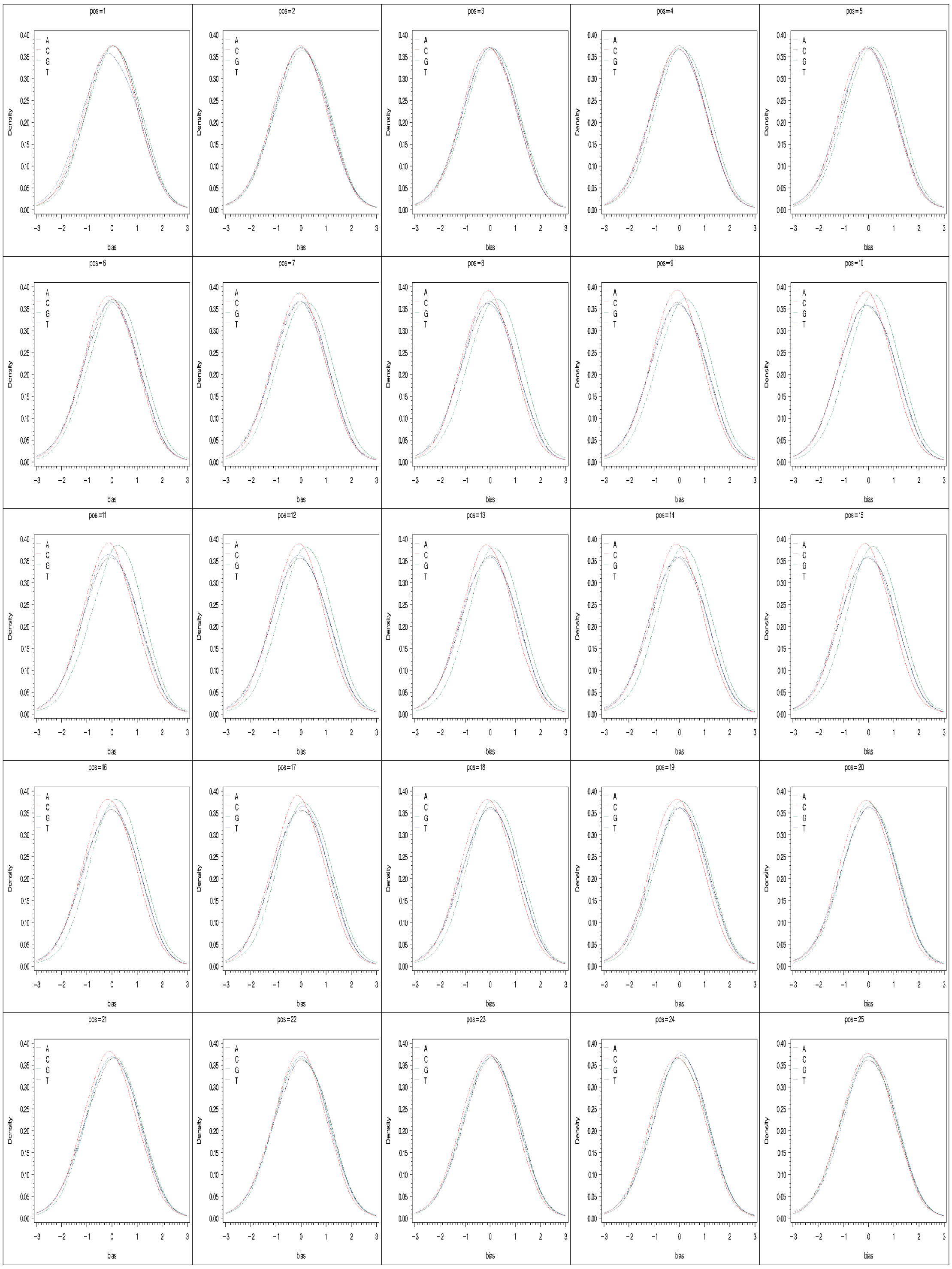

Histograms of the bias for each nucleotide, at each position along the probe, show normal distribution in all cases. Thus the mean values of each distribution can be used to formulate a sequence-based description of bias, in order to minimise noise in the data.
