# Supplementary material for: Custom Design and Analysis of High-Density Oligonucleotide Bacterial Tiling Microarrays
Source: PLoS One. 2009 Jun 17;4(6):e5943. doi: 10.1371/journal.pone.0005943 (PMC2691959; doi:10.1371/journal.pone.0005943)
Supplement: Table S1 — Probe density parameter overview (0.05 MB PDF) [file pone.0005943.s004.pdf]

Table S1. Probe density parameter overview

	***N.meningitidis* non-coding**	***N.meningitidis* coding**	***E.coli* non-coding**	***E.coli* coding**
**Minimum oligo spacing**	5	25	7	25
**Minimum total score**	0.2	0.2	0.4	0.4
**Max number of probes per region**	Unlimited	32	Unlimited	19
