# Supplementary material for: Custom Design and Analysis of High-Density Oligonucleotide Bacterial Tiling Microarrays
Source: PLoS One. 2009 Jun 17;4(6):e5943. doi: 10.1371/journal.pone.0005943 (PMC2691959; doi:10.1371/journal.pone.0005943)
Supplement: Table S2 — Initial OligoWiz 2.0 parameter settings (0.05 MB PDF) [file pone.0005943.s005.pdf]

Table S2. Initial OligoWiz 2.0 parameter settings

These settings were used for the initial selection of all possible probes.

Parameter	Value
OligoAimLen	25
OligoMaxLen	25
OligoMinLen	25
MinPercHom	75
MaxPercHom	98
MinHomLen	15
MaxHomFrac	0.8
Pseudocounts	5
PosScoreType	1 (Random)
DNA:RNAHyb	NO
AimTm	Find optimal Tm
