# Supplementary material for: Custom Design and Analysis of High-Density Oligonucleotide Bacterial Tiling Microarrays
Source: PLoS One. 2009 Jun 17;4(6):e5943. doi: 10.1371/journal.pone.0005943 (PMC2691959; doi:10.1371/journal.pone.0005943)
Supplement: Table S3 — Final OligoWiz 2.0 score-weight parameters (0.05 MB PDF) [file pone.0005943.s006.pdf]

Table S3. Final OligoWiz 2.0 score—weight parameters

Score type	Score weight
Cross-hybridization	4.0
Temperature model	0.3
Folding energy	1.0
Low complexity	1.0
Position	0.0
