# Supplementary material for: Custom Design and Analysis of High-Density Oligonucleotide Bacterial Tiling Microarrays
Source: PLoS One. 2009 Jun 17;4(6):e5943. doi: 10.1371/journal.pone.0005943 (PMC2691959; doi:10.1371/journal.pone.0005943)
Supplement: Table S4 — Overview of the control probes (0.07 MB PDF) [file pone.0005943.s007.pdf]

Table S4. Overview of the control-probes

**Probeset**	**Control of ***	**Replicates**
bioB, bioC, bioD, creX	Hybridization	7
lys, phe, thr, dap	Fragmentation and labelling	6
Hxb2 – Yeast<br>*trpnX*	Negative controls / background estimation	6
Custom set	Negative controls / background estimation	6

* This column describes what the control probes where used for in these experiments. The Hxb2-Yeast set would not be negative controls if any cDNA specific for these probes had been added to the hybridization cocktail. No specific RNA was added for the *trpnX* probeset, hence it became a negative control. (RNA for *trpnX* was previously added together with RNA for *lys, phe, thr and dap*, but has now been removed from the protocol)
