# Supplementary material for: Custom Design and Analysis of High-Density Oligonucleotide Bacterial Tiling Microarrays
Source: PLoS One. 2009 Jun 17;4(6):e5943. doi: 10.1371/journal.pone.0005943 (PMC2691959; doi:10.1371/journal.pone.0005943)
Supplement: Table S5 — Additive coefficients for sequence specific bias adjustments (0.07 MB PDF) [file pone.0005943.s008.pdf]

Table S5. Additive coefficients for sequence specific bias adjustments.

Position	Base	LSMEAN	STDERR
1	A	-0.130654065	0.0073277596
1	C	-0.179239647	0.0070064542
1	G	-0.071564317	0.0065946517
1	T	-0.1982988	0.007328418
2	A	-0.155531831	0.0072826193
2	C	-0.168881255	0.0071512619
2	G	-0.057360206	0.0065940872
2	T	-0.197983537	0.0074274186
3	A	-0.167190542	0.0071344239
3	C	-0.170298527	0.0071338047
3	G	-0.033881465	0.0066975081
3	T	-0.208386296	0.007377957
4	A	-0.174390193	0.0070918495
4	C	-0.175702579	0.0072208415
4	G	-0.018818317	0.0067633803
4	T	-0.210845741	0.0072263127
5	A	-0.163526757	0.0071052663
5	C	-0.193537856	0.0071718561
5	G	0.0029195145	0.0067192724
5	T	-0.225611731	0.007247853
6	A	-0.179063424	0.007111143
6	C	-0.204240598	0.0071466447
6	G	0.0219532116	0.0067102436
6	T	-0.218406019	0.0072266944
7	A	-0.17882535	0.0071776806
7	C	-0.207940867	0.0070996019
7	G	0.0387937858	0.0067043799
7	T	-0.231784398	0.0071801879
8	A	-0.179639089	0.0071108123
8	C	-0.228827246	0.0071158813
8	G	0.0587926637	0.0067316839
8	T	-0.230083158	0.0071643797
9	A	-0.188040335	0.0070837601
9	C	-0.23299724	0.0071497484
9	G	0.070295507	0.006740183
9	T	-0.229014762	0.0071776961
10	A	-0.186394346	0.0071146429
10	C	-0.246150874	0.0071764833
10	G	0.0722167555	0.0067541952
10	T	-0.219428366	0.0071279847
11	A	-0.187279208	0.0070922773
11	C	-0.252546308	0.0071305635
11	G	0.0857656767	0.0067454499
11	T	-0.225696991	0.0071975384
12	A	-0.198938204	0.0071467241
12	C	-0.238993354	0.0071590685
12	G	0.0825349662	0.0066771428
12	T	-0.224360237	0.0071903108
13	A	-0.19111176	0.0071244051
13	C	-0.248867573	0.0071456592
13	G	0.0851912982	0.0067400024

13	T	-0.224968795	0.0071910837
14	A	-0.191207728	0.0070703072
14	C	-0.242554014	0.0071586709
14	G	0.0803308305	0.0067401845
14	T	-0.226325917	0.0071988659
15	A	-0.178525929	0.0071135578
15	C	-0.244339534	0.0071305127
15	G	0.0659110854	0.0067283988
15	T	-0.222802453	0.0072105559
16	A	-0.199674281	0.0070909023
16	C	-0.228844786	0.0071561988
16	G	0.053764323	0.0067446647
16	T	-0.205002085	0.0072118729
17	A	-0.179233808	0.007118652
17	C	-0.225333594	0.0071183779
17	G	0.0258193798	0.006786502
17	T	-0.201008807	0.0071545015
18	A	-0.179517229	0.0071254345
18	C	-0.216750471	0.007145241
18	G	0.0124836131	0.0067081326
18	T	-0.195972743	0.0072065975
19	A	-0.152612291	0.0070897233
19	C	-0.213518262	0.007145709
19	G	-0.01890592	0.0067504513
19	T	-0.194720357	0.0072039968
20	A	-0.154092662	0.0070697766
20	C	-0.191582219	0.0071834
20	G	-0.049728731	0.006757411
20	T	-0.184353217	0.007182833
21	A	-0.160081569	0.0070843105
21	C	-0.179582733	0.007125882
21	G	-0.056474621	0.0067762584
21	T	-0.183617906	0.0071909407
22	A	-0.15001509	0.0070616327
22	C	-0.17043393	0.0071543366
22	G	-0.086161881	0.0068305793
22	T	-0.173145928	0.0071328304
23	A	-0.147656103	0.0071841219
23	C	-0.166238502	0.0071644535
23	G	-0.094898645	0.0067748921
23	T	-0.17096358	0.0070798523
24	A	-0.148973182	0.0071961048
24	C	-0.151328451	0.0070389601
24	G	-0.105733318	0.0067785111
24	T	-0.173721879	0.0072195371
25	A	-0.153473611	0.0070819679
25	C	-0.169973078	0.0070187204
25	G	-0.106244542	0.0067086306
25	T	-0.150065598	0.0072583141

These are the correction coefficients that might be applied to get an even more correct comparison between probes of different nucleotide compositions according to our GLM.
