# Supplementary material for: Custom Design and Analysis of High-Density Oligonucleotide Bacterial Tiling Microarrays
Source: PLoS One. 2009 Jun 17;4(6):e5943. doi: 10.1371/journal.pone.0005943 (PMC2691959; doi:10.1371/journal.pone.0005943)
Supplement: File S1 — Supplementary file with a thorough discussion about the quality assessments done on the presented normalisation method and comparisons to gcRMA, RMA and VSN. (3.30 MB PDF) [file pone.0005943.s009.pdf]

Supplementary_file_2.pdf

Normalization: why and how?

The issue of normalization is a many-faceted and vexing one. It is appealing to think that there should be an optimal method, which can be applied to a dataset that optimises the results for all further analyses.
Among the most accepted standard existing methods, gcRMA, RMA and VSN, which may be applied to the data, one might expect to find this "silver bullet".

The first question to be answered when it comes to normalization is how the method improves the data and, following on from that, how we can quantify the improvement by means of a test. Subordinate to this question is whether a normalization that improves the data for a specific analysis remains an improvement under other analysis conditions.

To illustrate this simplistically, sequence-based correction of tiling data may indeed reduce the noise measured along a single transcriptome, but in the case of a comparative analysis between a stressed and unstressed dataset, the normalisation has no value. This will be discussed in greater detail later.

Origins of noise: or why we need to normalize in the first place

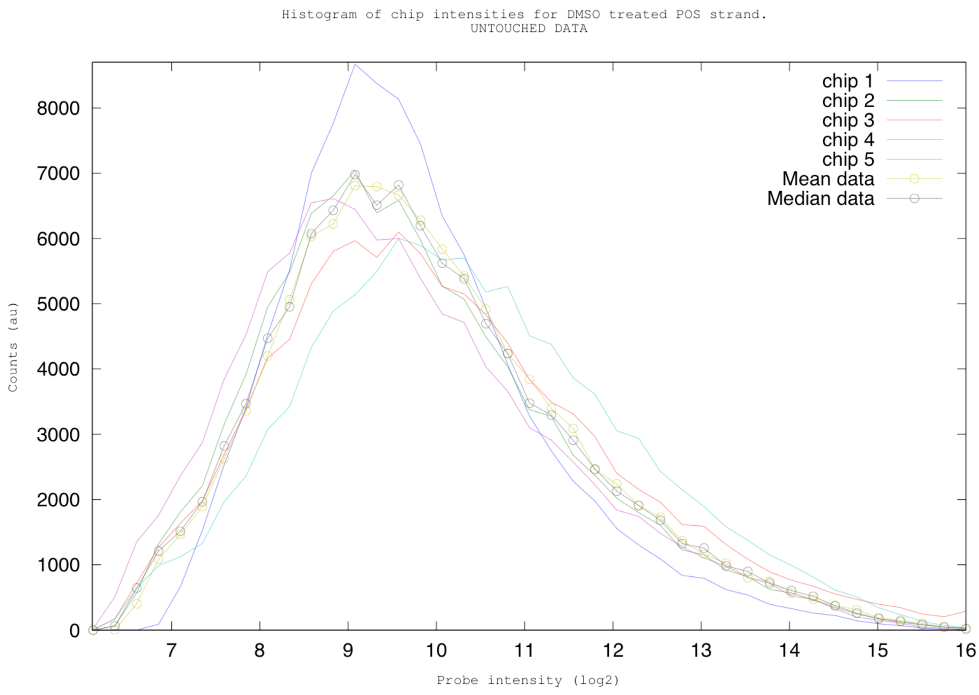

Figure 1

First let us consider the sources of noise in the data. A good overview of the variation in signal between chips can be achieved by plotting histograms of the intensity data from all 5 chips. Ideally, since all chips represent the same data, the 5 histograms should overlap closely. In practice, variation in preparation and scanning phases can induce notable systematic variation. **Fig. 1** shows the variation between 5 un-normalized chips for DMSO treated *E. coli*. This level of variation appears quite large, and in some cases is definitely the result of more than simple measurement variation around the same mean signal levels. This implies at very least a linear deviation in signal attributable to variation in scanning intensity.
Since probes are distributed randomly around the surface of the chip, a simple plot of probe intensity across individual chips gives little information. **Fig. 2** shows the probe intensities on chip 3 from the data shown in fig. 1, with non-probe and control-probe regions masked out. It is difficult to distinguish any systematic variation that could be induced by scanning.

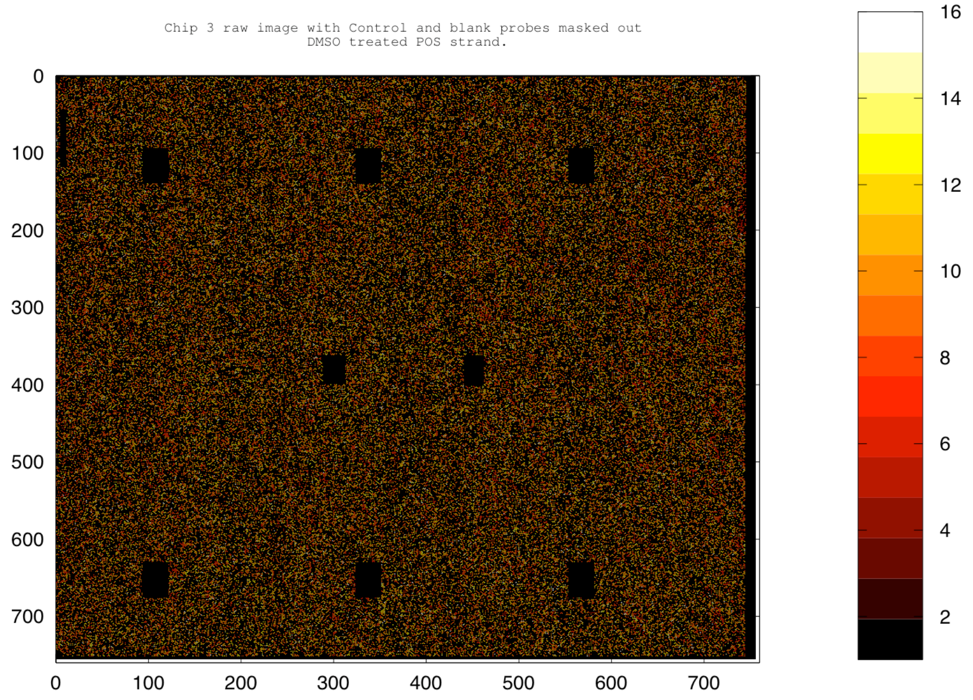

Figure 2

Averaging each point by the values of all probes contained in a surrounding window of half-width 50 points, allows a heatmap to be built showing local background intensity. Since each point is effectively calculated from a minimum of 498 neighbouring randomly-selected probes (average 1797 probes per point) one would expect to see very little variation across the chip, unless there are scanning-dependent fluctuations in the signal. **Fig. 3** shows the result of this approach applied to the chip shown in fig. 2. There is obvious "streaking" and a variation in background intensity between ~9.4 (dark red patches) and ~10.2 (pale yellow regions) implying a variation in background intensity of up to 0.8

where ideally none would be expected. These patterns are not consistently strongly correlated between chips.

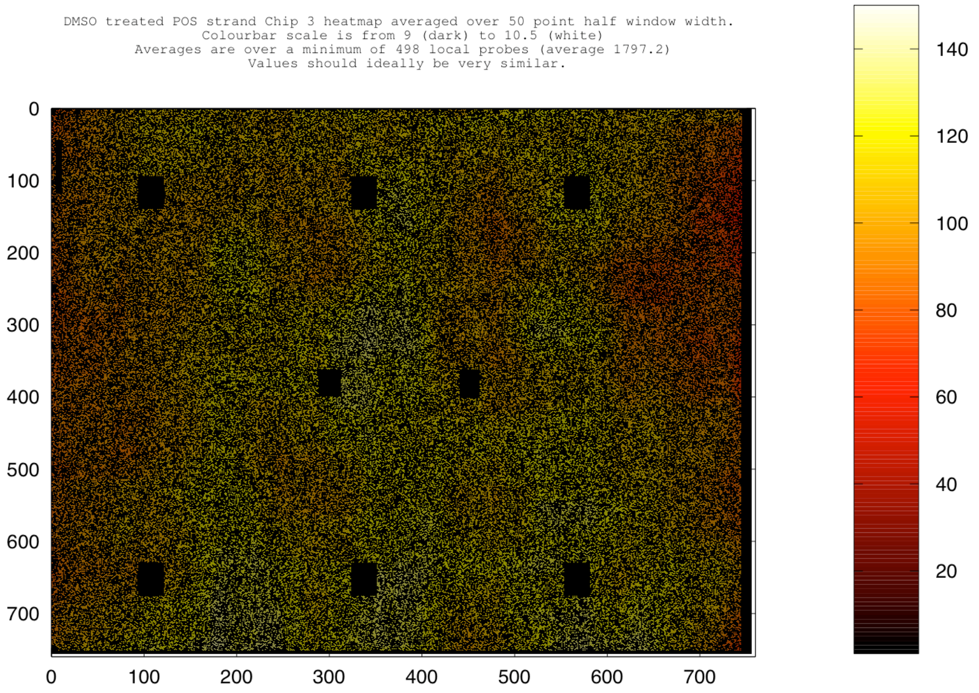

Figure 3

Thus we see that the variation between chips consists of a background baseline shift and (judging by the differing widths of individual histograms in fig. 1) variability in the presumed linearity of probe response to scanning. Hence, as is standard, any normalization algorithm would be expected to compensate for these variations with a chip-wise baseline shift and potentially a rescaling of probe intensities such that the histograms of probe intensities for individual chips overlap better.

Normalization: making data better

Now we must consider how best to quantify the effect of a normalization method. An ideal description would define improvement as an increase in the signal-to-noise ratio, which requires us to define both signal and noise as quantities.
In the case of a single record of transcription generated by the combined data from 5 identical chips, there are two major ways to consider signal and noise. The first and most general approach is to compare the signals between like probes across all chips. This allows us to assess a mean signal level and standard deviation between probes across all chips. We can represent this on a scatter plot of intensity vs standard deviation, Fig. 4. Ideally this would show a globally consistent trend, independent of intensity, but given the variable baselines and

differing linear responses of probes between chips, we see clearly from the trend (red) that there is some inconsistency with un-normalized data. Nevertheless it is possible to take an average level (green) to define the global standard deviation for the dataset (0.43603).

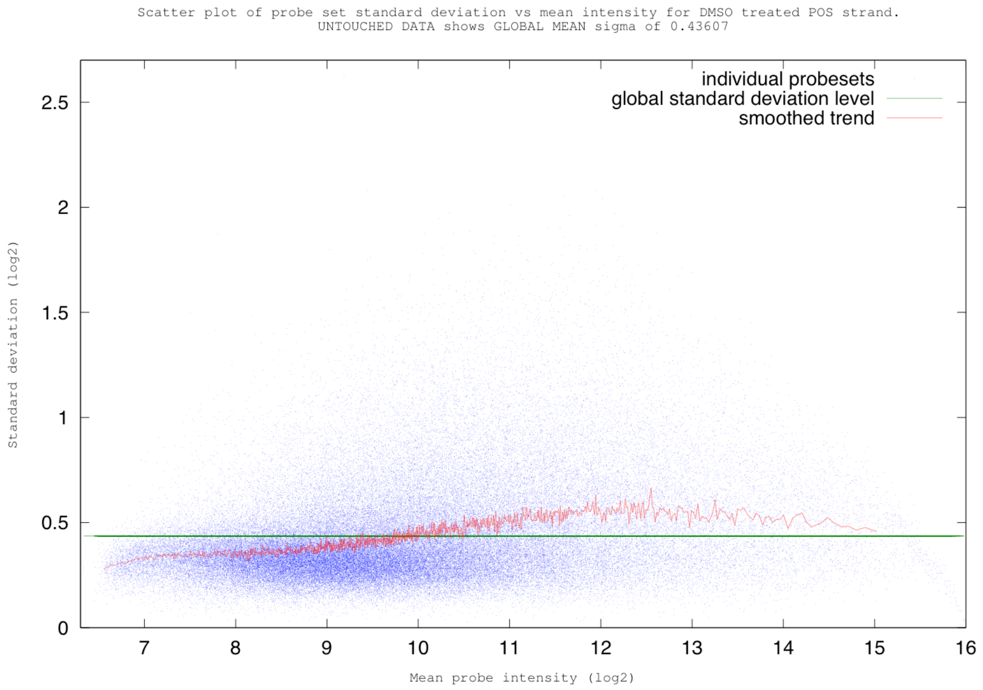

Figure 4

This global standard deviation (GSD) is a good general measure of the quality of any normalization routine. (Caveat: This is based upon the assumption that intensity levels from 5 probes are calculated by taking the mean rather than the median value of the 5 probes. The reasons for preferring a mean value will be discussed later in the text.) If the GSD is reduced by the method, it is likely that the normalization has improved the data.

A subsequent evaluation is applicable to well annotated data sets. On the assumption that all probes belonging to a single annotated region should be expressed equally, it is possible to calculate the average transcription level measured by all probes for each gene and thus measure the deviation of all coding probes from their local calculated mean. When compiled, this would be expected to show a single normal distribution of probes from their mean transcription levels as shown in Fig. 5. This is arguably the most sensitive method for assessing the quality of any normalization. Only methods that reduce the standard deviation below the level of raw data can be considered as candidate normalization approaches. Not only is this quality control applicable to single transcriptomes, but it can also be used on comparative (stressed vs unstressed) datasets.

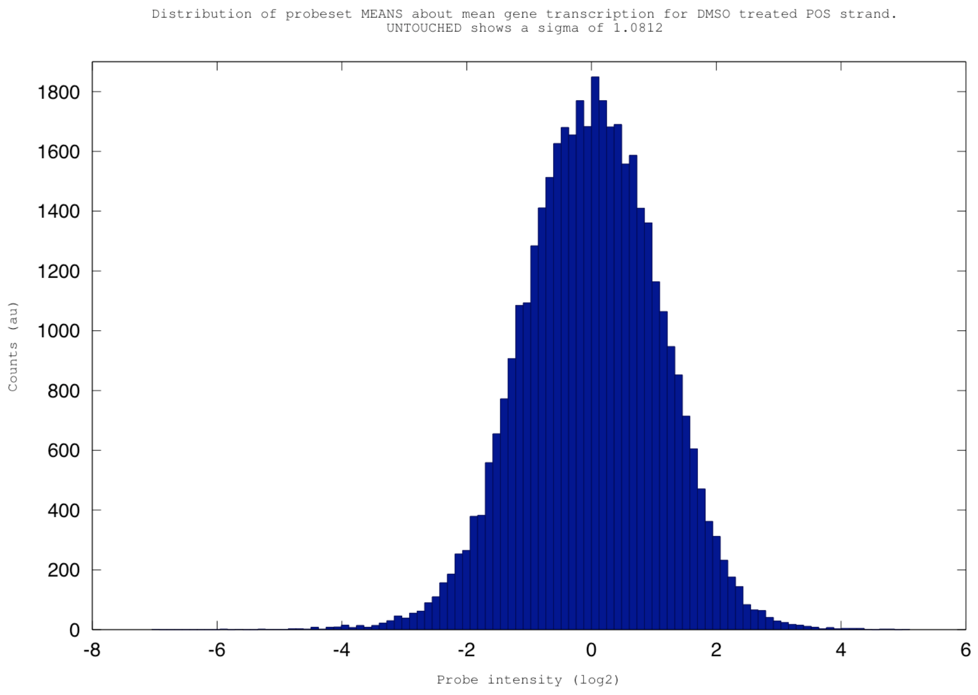

Figure 5

Thus this method can be considered to be the gold standard for assessing the overall applicability of a normalization routine. It is noteworthy that use of the median rather than mean values from a set of 5 probes consistently produces a higher standard deviation by this method, implying that use of the mean value is preferable to the median overall. Presumably this is dependent upon the quality of the data and one may expect that the median values return a better result with worse data where the variation between chips is larger or more irregular.

Final caveat: how the numbers might trick you

Now that we have defined two distinct methods for assessing the quality of any normalization, we must make one final check to ensure that the normalization is truly valid. The risk associated with any normalization method is that either some segment of the data is improved at the expense of another segment, or that the signals presented by the data are strongly deviated by the method, such that the results bear limited resemblance to the raw data, or are heavily overdamped. On the assumption that original data is relatively "good," one would require that normalization would not drastically affect the data; it would rather lightly tune the data. Any data that was so bad as to need serious rescaling would be of dubious quality and therefore questionable value.

Assessing the standard methods:

Having discussed the quality of normalized data, we must compare the quality of these methods to normalized data.
In the cases of both RMA and gcRMA, the algorithm struggles badly with the distribution of coding and non-coding probes that exist in our microarrays (a

constraint imposed upon us by the size of available chips and our specific interest in transcriptional variation in short non-coding regions in response to stress). Both methods therefore suffer in their attempt to divide probes up into transcribed or untranscribed distributions. The result in the case of RMA is a small decrease in GSD, but an increase in the gold standard variation around coding region, while gcRMA returned a large increase in both values. Thus primary analysis indicates that both methods are unsuitable. VSN by contrast shows a decrease in both values, which at first looks like an improvement in the data. However closer inspection shows that the dynamic range of the data is heavily reduced by the normalization, resulting in reduced overall signal and the removal of many known and validated differentially transcribed regions from comparative stressed vs unstressed measures. Thus we conclude that VSN is far too severe an algorithm since it fails to retain a significant portion of interesting data.
As a final evaluation, we show the mean values of all data points after normalization plotted against their corresponding unnormalized mean values in Fig. 6.

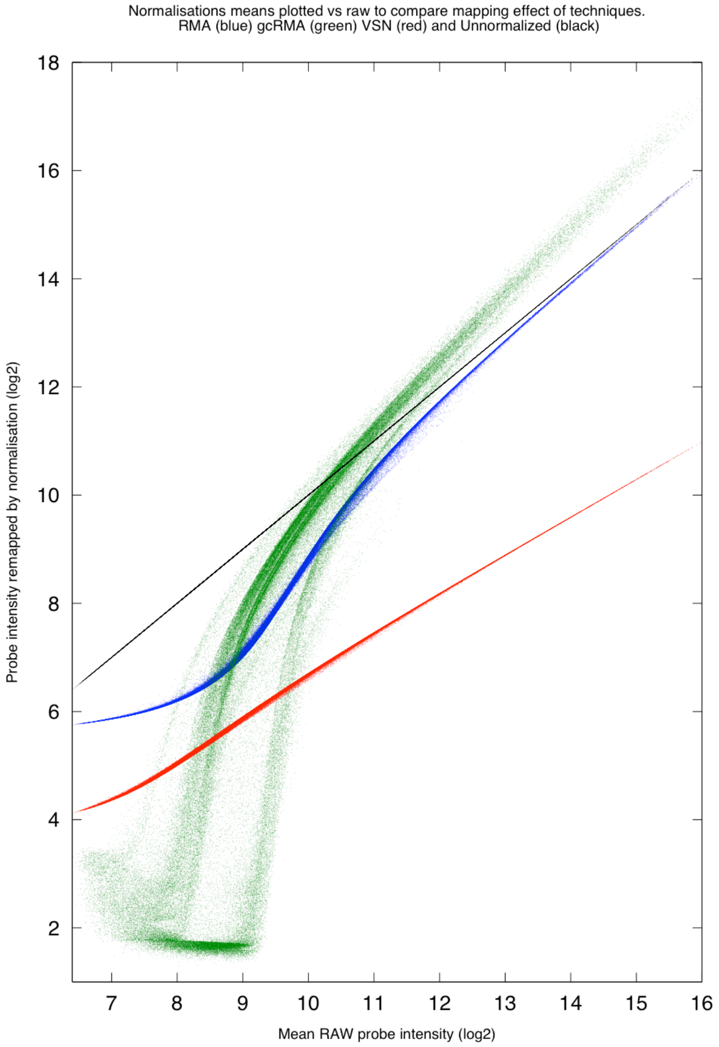

Figure 6

As previously declared, one would expect that a normalizing algorithm would not have a particularly severe effect upon good data, thereby inducing little deviation from the black line shown in fig. 6. (We can add to this that since we are unable to distinguish baseline transcription from background at a log2 probe intensity level below 9, that a normalisation routine that heavily deviated probe means below 9, but behaved well above this threshold; would be quite acceptable. However both RMA and gcRMA are seen to drag down probe means at values well above 9) Fig. 6 clearly shows that all three standard normalization routines act very heavily upon the data. In combination with the results of the first two tests of normalization quality, we can conclude that none of gcRMA, RMA or VSN returns an improvement over raw data.

Standard techniques don't work: what next?

Having concluded that none of the standard normalization routines work with our data, we are left to ask whether it is at all possible to renormalize the data by any method while fulfilling our criteria that define improved data.
One would intuitively expect that the components of standard methods, applied with a lighter touch would probably allow some improvement over raw data. This would imply, in the first instance, a baseline subtraction. In its simples form, this baseline subtraction consists of subtracting the mean probe intensity value for each chip, from every probe on the chip and immediately improves on the GSD value compared to raw data. The gold standard measure is not seen to change. What we see in Fig. 7, however, is that relatively few probes contribute extreme values of standard deviation, which hold the GSD value below but in the vicinity of the GSD shown in fig. 4.

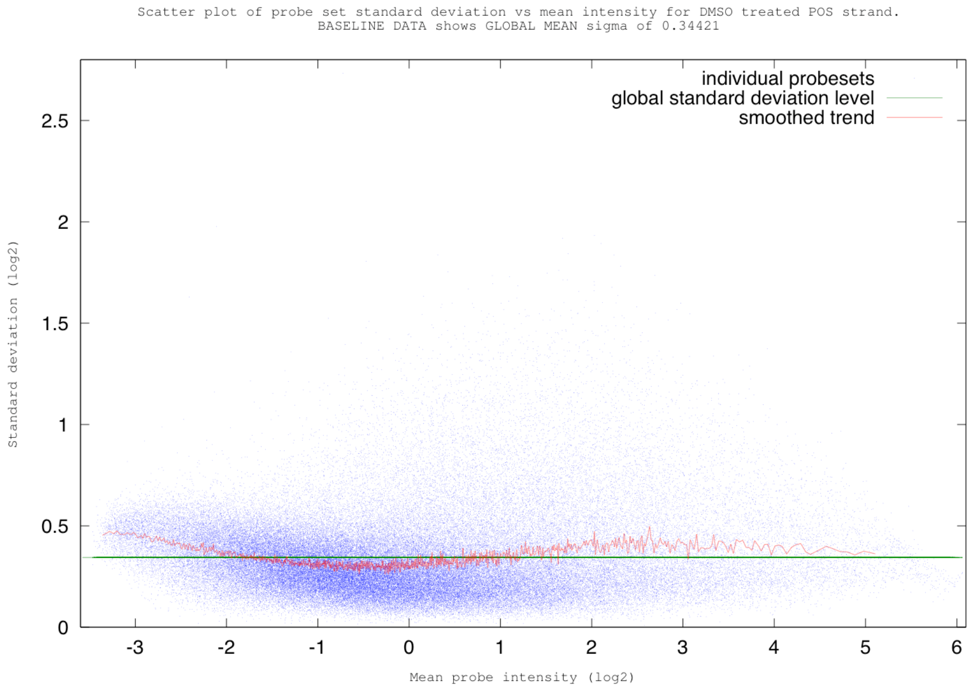

Figure 7

These extreme values are generally contributed by one or occasionally two probes that lie a long way from the remaining probes in the set. Use of a median value to define the overall probe intensity would therefore remove the majority of this problem, but as discussed earlier, in regard to our gold standard test, general use of the median produces more noise than use of the mean. Thus we took a different approach, whereby we could define these few points as outliers and remove them from the probe set. In order to find outliers, we sorted our probeset into ascending order and calculated the mean of the middle three probes. Taking the measured GSD, we can define threshold cutoffs 3GSDs above and below the central mean, beyond which any point has a greater than 99% chance of being an outlier. If a point is found to be an outlier by these criteria, we discard it, taking the mean value of the retained points as the mean value of the probeset. Fig. 8 shows the further improvement this makes upon the GSD value with the removal of just 10023 probes from a total of 526,750 (ie <2%).

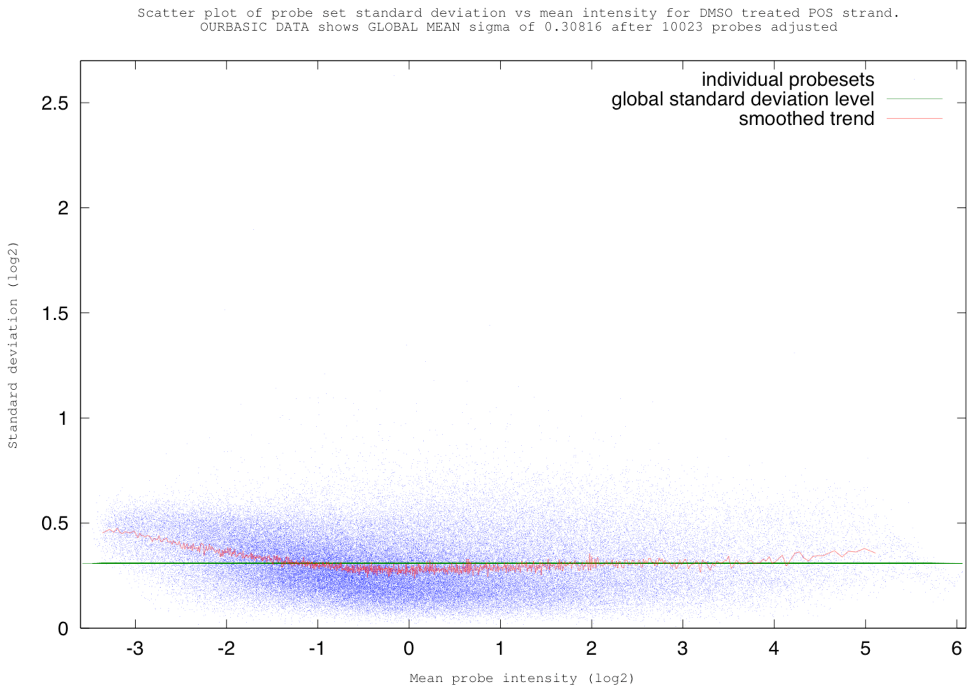

Figure 8

There is concurrently a small decrease in the standard deviation for the gold standard measure in 3 out of 4 cases, while the gold standard for the comparative data is slightly higher, although still lower overall than the gold standard value for raw data if median values are used instead of means.

Thus we conclude that this is the simplest and most transparent normalisation method we can apply which reduces the noise in the data without imposing significant variations upon it. It is here that we are obliged to conclude that no single method yields the best possible signal-to-noise ratio for all data. For the sake of consistency and clarity it is therefore necessary to apply the most broadly beneficial method.

We have investigated the value of additionally rescaling the data, such that all distributions are alike, but it is clear from analysis of the results that neither a simple linear rescaling of individual chips, nor a quantile-based rescaling can consistently reduce noise levels in the data to below the level seen in raw data.

It should be noted that a baseline subtraction for each probe using the local value calculated in the heatmaps, is somewhat better than a simple subtraction of individual chip means. However this does not outperform our outlier removal method.

We speculate that a highly complex analysis based around the assumption that probe signal is also dependent upon the local background intensity for individual chips, may allow for a normalization algorithm that is absolutely optimal. However, the small possible improvement to be gained from this was judged to be of insufficient value when compared to the effort required to understand whether convolving probe intensity with background signal intensity from heatmaps would actually yield significantly improved data.
Given time constraints and the wealth of biologically relevant results returned by our simple methods, we have opted to use data that is minimally normalised.
